# A comprehensive multivariate analysis of the center of pressure during quiet standing in patients with Parkinson's disease

**DOI:** 10.1186/s12984-024-01358-1

**Published:** 2024-04-23

**Authors:** Shintaro Fujii, Yusaku Takamura, Koki Ikuno, Shu Morioka, Noritaka Kawashima

**Affiliations:** 1 Graduate School of Health Sciences, Kio University, Nara, Japan; 2 Department of Rehabilitation, Nishiyamato Rehabilitation Hospital, Nara, Japan; 3 Department of Rehabilitation for Movement Functions, Research Institute of National Rehabilitation Center for Persons with Disabilities, 4-1 Namiki, Tokorozawa, Saitama 359-0042 Japan; 4 Neurorehabilitation Research Center, Kio University, Nara, Japan

**Keywords:** Parkinson’s disease, Quiet standing, Center of pressure, Exploratory factor analysis, Cluster analysis

## Abstract

**Background:**

We hypothesized that postural instability observed in individuals with Parkinson's disease (PD) can be classified as distinct subtypes based on comprehensive analyses of various evaluated parameters obtained from time-series of center of pressure (CoP) data during quiet standing. The aim of this study was to characterize the postural control patterns in PD patients by performing an exploratory factor analysis and subsequent cluster analysis using CoP time-series data during quiet standing.

**Methods:**

127 PD patients, 47 aged 65 years or older healthy older adults, and 71 healthy young adults participated in this study. Subjects maintain quiet standing for 30 s on a force platform and 23 variables were calculated from the measured CoP time-series data. Exploratory factor analysis and cluster analysis with a Gaussian mixture model using factors were performed on each variable to classify subgroups based on differences in characteristics of postural instability in PD.

**Results:**

The factor analysis identified five factors (magnitude of sway, medio-lateral frequency, anterio-posterior frequency, component of high frequency, and closed-loop control). Based on the five extracted factors, six distinct subtypes were identified, which can be considered as subtypes of distinct manifestations of postural disorders in PD patients. Factor loading scores for the clinical classifications (younger, older, and PD severity) overlapped, but the cluster classification scores were clearly separated.

**Conclusions:**

The cluster categorization clearly identifies symptom-dependent differences in the characteristics of the CoP, suggesting that the detected clusters can be regarded as subtypes of distinct manifestations of postural disorders in patients with PD.

## Introduction

Parkinson's disease (PD) is associated mainly with dysfunction of the basal ganglia, resulting in motor symptoms such as tremor, rigidity, bradykinesia, postural instability, gait disturbance (PIGD). Although postural instability becomes more prominent with the progression of PD symptoms [1], postural sway has been shown to be abnormal even in the early stages of PD with mild motor symptoms [2, 3]. Since postural instability in PD patients is associated with risks of falling [4], injury [5], a reduced quality of life [6], it is quite important to find ways to precisely evaluate postural instability and improve postural control strategies in individuals with PD.

In the clinical evaluation of PD patients, a component of the Unified Parkinson's Disease Rating Scale (UPDRS), i.e., the pull test, is commonly used to assess postural instability. The definition of postural instability is a score of three or higher, which categorizes the stage of the examine by the Hoehn and Yahr (HY) scale as stage 3 [7]. However, the pull test is not sufficient to understand the characteristics of postural disturbance [8, 9]. The center of pressure (CoP), recorded as a trajectory in the anterior–posterior and medial–lateral planes, has also been used as an evaluation tool for postural disorders including those in PD [10, 11]*.* However, the CoP findings obtained from PD patients have been controversial. For example, some studies have shown increased postural sway [12, 13], but others reported a small range and a slow component of CoP displacements [14, 15]*.* Although many variables can be calculated from CoP time-series data, i.e., the displacement, the velocity, and the frequency of sway, it is not easy to interpret these variables as consistent factors that cause postural instabilities [16]. Considering the diversity of postural instability in PD patients, it is necessary to conduct comprehensive analyses based on various evaluated parameters obtained from CoP data during standing.

**Fig. 1:**
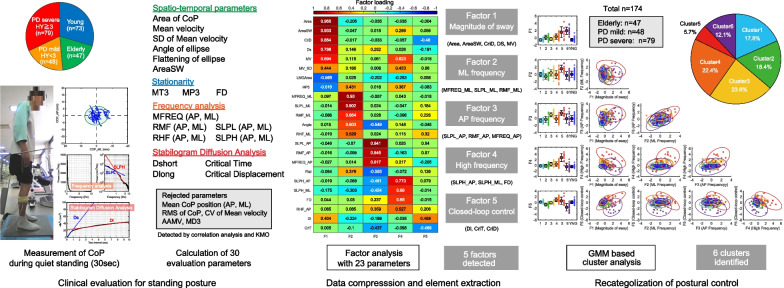
Framework of the study. Left panel: The experimental set-up and the variables calculated from the CoP time-series data. Upper left pie chart shows the percentage of subjects in each clinical categorization. Right shows 23 variables used in the analysis and 17 variables rejected. Middle panel: Summary of the results obtained by the exploratory factor analysis (EFA). The color matrix shows the results of the factor loadings of each variable on the extracted factors. F1: magnitude of sway, F2: ML frequency component, F3: AP frequency component, F4: high-frequency component, F5: closed-loop control. Right panel: Comparison of factor scores by cluster in a Gaussian mixture model (GMM)-based cluster analysis. *p < 0.05, **p < 0.01 by post-hoc test (Steel–Dwass test) and Kruskal–Wallis test. Scatterplots shows the EFA in each cluster with 95% confidence ellipsoids and ellipses. Upper right pie chart: the percentage of each cluster

Physicians and therapists well recognize that there are different subtypes in PD comprised of different contributions of PD-related symptoms, clinical signs, the medical history, age at onset, the rate of disease progression, and more [17, 18]. As shown in Fig. 1, we performed factor analysis based on 23 variables that were calculated from CoP time-series data. We hypothesized that the factor analysis process could effectively reveal distinct components of postural instabilities in PD patients. Given that the pathophysiology of postural instability in PD is complex and may be a combination of disease factors due to PD and compensatory factors involving aging effects [19], we included both young and elderly healthy individuals' data in the multivariate component analysis. We then performed a cluster analysis with a Gaussian mixture model (GMM) using the factor loading score for the identification for subtypes of postural instability in PD.

To the best of our knowledge, this is the first study to characterize postural instability in PD by performing a comprehensive multivariate analysis based on various evaluated parameters obtained from CoP time series data during quiet standing. We selected various aspects of analytical way for the postural control, for example, spatio-temporal parameters, frequency domain, and time-series fluctuation analysis, etc. Since the extracted factors obtained from exploratory factor analysis (EFA) were the result of data compression and multivariate analysis, those can be regarded as consisting elements of postural control in PD patients. Our factor analysis revealed five distinct elements of postural disturbance in PD patients, and a cluster analysis then identified six PD subtypes that consist of different contributions of those five elements. Presumably because the high quality of the selection of parameters and data compression process, cluster classification based on factor scores clearly identified symptom-dependent differences among PD patients.

## Materials and methods

### Participants

The participants were 138 PD patients (age 71.1 ± 8.1 years, time from onset of PD 6.7 ± 4.9 years) from three collaborating hospitals, and 51 healthy older adults (elderly: age 72.6 ± 5.6 years) aged 65 years or older and 73 healthy young adults (young: age 28.6 ± 5.2 years). Data recordings of the PD patients were performed as clinical routine of postural assessments at each collaborating hospital while those of the elderly and the young subjects were recruited among community livers around Kio University. Participants who had a history of cerebrovascular or spinal cord disease or musculoskeletal disease, or difficulty holding a quiet standing position for 30 s without assistance were excluded. Data for 11 PD patients, four elderly and two young adults were removed by outlier analysis, resulting in a final analysis of 127 PD patients (age 71.2 ± 8.2 years, time from onset of PD 6.6 ± 4.7 years), 47 elderly (age 72.4 ± 5.4 years), and 71 young adults (age 28.2 ± 5.1 years).

The Unified Parkinson's Disease Rating Scale (UPDRS) motor section [20] and HY stage [7] were used as clinical scores for PD symptoms at the time of study participation. We then classified the PD patients into the following two groups based on the severity/HY stage: (1) the mild PD group (PD_mild_ n = 48), where were the patients with an HY stage < 3 (dysfunction of the postural reflexes by pull test is negative); and (2) the severe PD group (PD_severe_: n = 79), i.e., the patients with HY stage ≥ 3 (dysfunction of the postural reflexes by pull test is positive). Each item in the UPDRS motor section was rated from 0 (normal) to 4 (severe), and the following sub-score items were assessed [21]: PIGD, items 27–30; Tremor, items 20 and 21; Rigidity, items 22a–22e; and Bradykinesia, items 23–26 and 31. The UPDRS and HY stage of the patient population were scored by well-trained clinicians of each collaborating hospital. In case of patients who prescribed levodopa were evaluated at an active state after several hours of regular medication, and patients who did not prescribed levodopa were measured regardless of the effect of the medication. Therefore, all patients were evaluated with their own averaged state.

### Experimental procedures and data analysis

The experimental procedure is depicted in Fig. 1. The participant stood on a force platform (BASYS, Tec Gihan Co., Kyoto, Japan). During the measurement, the participant was instructed to place both upper limbs on the lateral side of the trunk and to gaze at an indicator placed 2 m in front of him/her. Data recordings (sampling frequency: 1 kHz) were performed by the experienced therapists of three hospitals. All these stuffs had been well trained not only for the data acquisition, but also the instruction for the participant at the measurement. The center of pressure (CoP) time series data were processed by a fourth-order Butterworth filter with a low-pass filter (cut-off frequency 10 Hz). A total of 30 variables were calculated from the CoP, including spatial variables to estimate the area covered by the CoP and temporal variables to indicate the speed of the participant's sway (Table 1).

**Table 1:** Characteristics of each variable

Variable	Description
MeanPos. AP/ML	Mean position of sway, cm
RMS	Root mean square distance of CoP, cm
Area	Area of 95% confidence ellipse, cm^2^
Angle	Absolute value of angle between major axis and ML axis, degree
Flat	Flattening of 95% confidence ellipse
AreaSW	Mean triangle area enclosed by mean CoP position and two consecutive points, cm^2^
LNGArea	Total path length of CoP trajectory divided by 95% confidence ellipse
MV	Mean CoP velocity, cm/sec
MV_SD	Standard deviation of MV
MV_CV	Coefficient of variation of MV
AAMV	Average absolute maximal velocity, cm/sec
MFreq, AP/ML	Mean frequency of a circular motion with a radius equal to mean amplitude, Hz
RMF, AP/ML	Ratio of mid-frequency component (0.3–1 Hz) to total power value (0–10 Hz), %
RHF, AP/ML	Ratio of high frequency components (1–3 Hz) to total power value (0–10 Hz), %
SLPL, AP/ML	Linear regression against 0.15–1 Hz band in PSD log plot
SLPH, AP/ML	Linear regression against 1–5 Hz band in PSD log plot
MT3	Mean time interval between successive peaks on sway density curve at 3-mm radius, sec
MP3	Mean peak value on sway density curve at 3-mm radius, sec
MD3	Mean distance between in AP-ML plane successive peaks on sway density curve at 3-mm radius, mm
FD	Fractal dimension of CoP of planar movement
Ds	Slope of short-time region in SDA
Dl	Slope of long-time region in SDA
CriT	Time interval at the intersection of two regression lines on SDA
CriD	Mean square value at CriT on SDA

In this study, we used a single set of 30 s CoP data that is recorded as a part of routine clinical assessment. While multiple trials are recommended to calculate CoP parameters [22, 23], long-time measurements with several repetitions are not easy for the patient population, especially in cases of severe postural instability [24, 25]. Importantly, previous studied confirmed the validity and reliability of CoP variables calculated by a single 30-s data in subacute [26] and chronic stroke patients [27].

The following 12 variables were calculated as spatio-temporal parameters: (1, 2) the mean positions of the anterior–posterior direction (MeanPosAP) and the medio-lateral direction (MeanPosML), (3) the root mean square distance of the CoP (RMS), (4) the 95% confidence ellipse area (Area), (5) the angle of the ellipse area (Angle), (6) the flattening of ellipse area (Flat), (7) the mean triangle area enclosed by the mean CoP position and two consecutive points (AreaSW), (8) the total path length of the CoP trajectory divided by the 95% confidence ellipse (LNGArea), (9) the mean velocity (MV), (10) the standard deviation of velocity, (11) the coefficient of variation of velocity, and (12) the average absolute maximal velocity [28].

The following four variables were calculated as variables related to the stationarity of the CoP: (1) the mean time interval between successive peaks on the sway density curve at a radius of 3 mm (MT3) [29], (2) the mean peak value on the sway density curve at a 3-mm radius (MP3), (3) the mean distance between successive peaks in the AP-ML plane on the sway density curve at a 3-mm radius (MD3), and (4) the fractal dimension of the CoP of planar movement (FD).

Regarding the variables for the frequency analysis, we calculated 10 variables in the range of 0–10 Hz by using fast Fourier transformation after removing the mean value of the CoP time series. The frequency bands were classified as follows: 0.15–0.3 Hz for the low frequency, 0.3–1 Hz for the mid-frequency, and 1–3 Hz for the high frequency [30]. The low-frequency range is associated with visual control; the mid-frequency range is associated with vestibular and somatosensory information, and the high-frequency range is associated with intrinsic sensation and muscle activity [31]. We divided the power of each frequency band by the total power value up to 10 Hz.

Since the ratio of low and mid-frequencies showed a strong relationship, we used the mid-frequency (RMF) and high-frequency (RHF) variables. The scaling exponents of the power law-shaped power spectral density (PSD) in the frequency bands were characterized by linear regression against the 0.15–1 Hz (SLPL_AP/ML) and 1–5 Hz (SLPH_AP/ML) bands of the calculated PSD log plot [32]. The mean frequency (MFreq_AP/ML) was calculated as a circular motion of the same radius as the mean amplitude [33].

We performed a stabilogram diffusion analysis (SDA) for the CoP displacement in the planar directions [34]. The CoP SDAs were calculated by using the following equation:

$$<\Delta {x}^{2}>=\langle [x(t+\Delta t)-x(t){]}^{2}\rangle$$

where ‹∆x^2^› indicates the calculation of the mean of the time series. The calculation is repeated with increasing values of Δt in the range of 0–10 s. The resulting diffusion plot shows the mean squared displacement against the time interval Δt. The short-term (Ds) and long-term (Dl) diffusion coefficients were determined by linear regression on the diffusion plot. Ds and Dl correspond to one-half the slope of the respective linear fit to the SDA. The critical time (CriT) and critical displacement (CriD) were calculated from the linear intersection of the short- and long-term domains. The method of Amoud et al. was used to identify the slope [35]. In some cases, the fitting was manually modified to maintain a good-quality linear fit of the data. MATLAB software (ver. 2018a Mathworks, Natick, MA) was used for all analyses. Concerning the data reliability in diffusion coefficients in SDA, previous study already confirmed those even with a single measurement of CoP at least 30 s under quiet standing [36].

### Exploratory factor analysis

Before we conducted the exploratory factor analysis (EFA), we performed outlier removal using Hotelling's T-square method, i.e., the statistically abnormal detection by χ^2^-distribution [37], and then we removed 17 variables with p < 0.05 with the Bonferroni test (i.e., 0.05/n = 0.000190). Hotelling's T-square was calculated using the "pca" function in the MATLAB software.

The EFA was then conducted to evaluate the contribution of each CoP variable. The variable selection for the EFA was performed using correlation criteria, i.e., Pearson's correlation as 0.3 ≤ r ≤ 0.9, and Kaiser–Meyer–Olkin (KMO) criteria, i.e., the measure of sampling adequacy (MSA) for individual variables in the KMO test at ≥ 0.5. The selected variables were used in the subsequent analysis after transformation to z-score. The robust maximum likelihood (MLR) with oblique geomin rotation was used for factor extraction. The number of factors was determined as the number with the smallest Bayesian information criterion (BIC) value among the number of factors estimated by a parallel analysis. If any variables with factor loadings < 0.4 were included (factor loading-based criteria), these variables were excluded and then the factor analysis was performed again.

### Statistical analysis

Since the normality of the variables was not confirmed by the Shapiro–Wilk test (p < 0.05), the significance of differences in the patients' clinical scores and factor scores for posturography variables among the groups was examined by the Mann–Whitney U-test (comparison between PD groups) or the Kruskal–Wallis test, and then a post-hoc test (corrected for multiple comparison by the Steel–Dwass test) was applied for the comparisons of pairs of groups. Spearman's rank correlation coefficient was used to correlate the calculated factor scores with the UPDRS and the duration of disease.

To classify subgroups based on differences in the characteristics of postural instability in PD, we performed a cluster analysis with a Gaussian mixture model (GMM) using a factor score. GMM clustering is a probabilistic model-based clustering method and is a more robust method than other clustering methods [38]. In this clustering approach, the numbers of clusters and differences in the distribution and its volume, shape, and orientation can also be compared with statistical information criteria such as the BIC and/or the integrated complete-data likelihood criterion (ICL) [39]. We defined the following criteria for the number of clusters and distribution features: the number of clusters is 4 to 30 (the aim was to provide a comparable or more detailed classification than the categories of Young, Elderly, PD_mild_, and PD_severe_). The model with high theoretical validity with better BIC and ICL values was selected as the optimal model from among several models that met these criteria. All clustering procedures were performed by the program R ver. 3.5.0. We used the mclust5 package (an add-on package in R) for GMM clustering. The data are presented as the mean ± standard deviation. Significance was accepted at p < 0.05.

## Results

The results of the clinical evaluations are summarized in Table 2. The UPDRS motor symptoms were significantly higher in the PD_severe_ group (n = 79) compared to the PD_mild_ group (n = 48) for the total score (PD_mild_: 17.7 ± 9.9, PD_severe_: 30.1 ± 12.4; W = 816, p < 0.01), rigidity (PD_mild_: 4.4 ± 3.2, PD_severe_: 6.5 ± 3.5; W = 1231, p < 0.01), PIGD (PD_mild_: 3.5 ± 2.5, PD_severe_: 6.8 ± 2.7; W = 687, p < 0.01), and bradykinesia (PD_mild_: 7.4 ± 5.6, PD_severe_: 12.5 ± 6.0; W = 953.5, p < 0.01). There was no significant between-group difference in tremor score (PD_mild_: 1.2 ± 1.5, PD_severe_: 2.2 ± 2.9; W = 1588, p = 0.11). The patient's age and the duration of disease were nearly the same in the PD_mild_ and PD_severe_ groups (age: PD_mild_: 70.8 ± 8.4, PD_severe_: 71.4 ± 8.1, W = 1784, p = 0.58; duration of disease: PD_mild_: 6.1 ± 3.8, PD_severe_: 7.0 ± 5.1, W = 1776, p = 0.63).

**Table 2:** Characteristics of the patients with PD and the healthy controls

	Classification by severity					Classification by Cluster						
	YNG	ELD	PD_mild_	PD_severe_	Statistics	Cluster 1	Cluster 2	Cluster 3	Cluster 4	Cluster 5	Cluster 6	Statistics
	n = 71	n = 47	n = 48	n = 79		n = 31	n = 32	n = 41	n = 39	n = 10	n = 21	
Male/female	35/36	17/30	27/21	32/47	ns	11/20	12/20	20/21	18/21	3/7	12/9	ns
Age, yrs	28.5 ± 5.1	72.4 ± 5.4	70.8 ± 8.4	71.4 ± 8.1	YNG vs. ELD, PD_mild_, PD_severe_**	71.1 ± 7.2	70.6 ± 8.1	73.2 ± 6.1	71.3 ± 7.5	74.7 ± 7.6	69.1 ± 7.8	ns
Disease duration, yrs	–	–	6.1 ± 3.8	7.0 ± 5.1	ns	3.9 ± 3.4	7.0 ± 5.2	5.9 ± 3.5	7.6 ± 5.2	7.0 ± 5.3	8.4 ± 4.4	C1 vs. 4, 6*
Age at onset, yrs	–	–	64.7 ± 9.3	64.3 ± 8.9	ns	66.9 ± 7.6	62.5 ± 10.3	67.4 ± 8.8	64.0 ± 9.1	67.7 ± 10.5	59.0 ± 6.0	C1 vs. 6* C3 vs. 6**
UPDRS Total	–	–	17.7 ± 9.9	30.1 ± 12.4	**	25.2 ± 13.2	23.2 ± 14.4	28.3 ± 11.2	28.3 ± 14.6	27.9 ± 8.8	17.5 ± 10.0	C3 vs. 6*
UPDRS Rigidity	–	–	4.4 ± 3.2	6.5 ± 3.5	**	5.6 ± 3.2	4.7 ± 3.7	7.9 ± 2.9	6.2 ± 3.5	5.3 ± 3.7	3.2 ± 2.6	C2 vs. 3* C3 vs. 6** C4 vs. 6*
UPDRS PIGD	–	–	3.5 ± 2.5	6.8 ± 2.7	**	5.0 ± 2.6	4.7 ± 2.7	6.4 ± 3.8	6.0 ± 3.0	6.1 ± 3.1	4.8 ± 2.6	ns
UPDRS Bradykinesia	–	–	7.4 ± 5.6	12.5 ± 6.0	**	10.8 ± 7.0	10.3 ± 6.2	10.8 ± 5.5	11.8 ± 7.4	12.4 ± 4.4	7.3 ± 5.0	ns
UPDRS Tremor	–	–	1.2 ± 1.5	2.2 ± 2.9	ns	1.8 ± 2.5	2.3 ± 3.1	1.3 ± 1.7	2.2 ± 2.5	2.1 ± 2.6	1.3 ± 2.5	ns

Data are mean ± SD. *p < 0.05, **p < 0.01. *YNG* young, *ELD* elderly, *ns* nonsignificant, *PD*_*mild*_ Parkinson's disease-mild, *PD*_*severe*_: Parkinson's disease-severe, *PIGD* postural instability and gait disturbance, *UPDRS* The Unified Parkinson's Disease Rating Scale

### Exploratory factor analysis

To extract distinct components of the postural instabilities in the PD patients, we performed an exploratory factor analysis (EFA) with 23 variables calculated from the patients' CoP time-series data. Four variables and a single variable were removed in the correlation and KMO criteria, respectively (MeanPos_AP, RMS, AAMV, MD3, and MV_CV). In addition, two variables were removed in the factor loading criteria (MeanPos_ML and MT3). A total of five factors were extracted by the EFA with 23 accepted variables (Fig. 1).

Figure 1's middle panel depicts the loading score of each variable on the detected factors. Each factor can be interpreted as follows: F1 was interpreted as a "magnitude of sway factor," with large contributions such as Area, MV, and Ds in the SDA. F2 was interpreted as a "ML frequency component factor," with large contributions from MFreq_ML and SLPL_ML. In contrast, F3 was interpreted as an "AP frequency component factor" with large contributions from MFreq_AP and SLPL_AP. F4 was interpreted as a "high-frequency component factor" with large contributions from SLPH_AP and ML and FD. F5 was interpreted as a "closed-loop control factor," with large contributions from Dl and CriT in SDA.

**Fig. 2:**
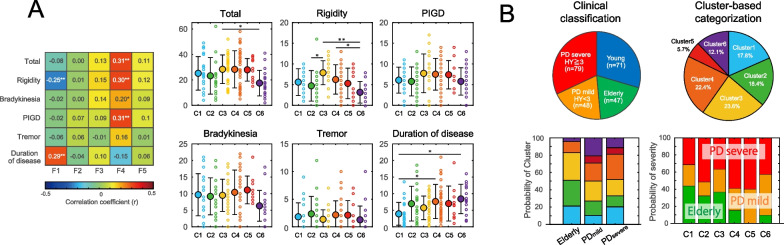
Clinical characteristics. **A** Color matrix: indicating the correlation coefficient between factor scores and UPDRS scores and the duration of disease. *p < 0.05, **p < 0.01 by Spearman's rank correlation coefficient. Plot graphs: the results of the comparison of clinical evaluations in each cluster. Data are mean ± SD. *p < 0.05, **p < 0.01 by post hoc test (Steel–Dwass test) and Kruskal–Wallis test. **B** Percentages of members. Left panel: The percentages of Elderly, PD_mild_ and PD_severe_ in each cluster. Percentages of the clusters in the clinical classification. Right panel: The percentages of clinical classification in each cluster

The relationship between factor scores and UPDRS scores and the duration of disease are shown as a color matrix in Fig. 2A. F1 showed a significant positive correlation with the disease duration (ρ = 0.29, p < 0.01) but a significant negative correlation with UPDRS rigidity (ρ =  − 0.25, p < 0.01). F4 was positively correlated with the UPDRS total score (ρ = 0.31, p < 0.01), rigidity (ρ = 0.30, p < 0.01), bradykinesia (ρ = 0.20, p = 0.02), and PIGD (ρ = 0.31, p < 0.01). F2, F3, and F5 showed no significant correlations.

### Gaussian mixture model-based cluster analysis

Since the comparison of factor scores confirmed that the Young healthy group had postural control characteristics that were distinctly different from those of the Elderly healthy and PD groups, we performed GMM-based clustering on the data from the healthy Elderly and PD patients (n = 174). The GMM-based cluster analysis classified the six clusters.

As shown in Fig. 2B, 31 patients (17.8%) were classified as Cluster 1, 32 patients (18.4%) as Cluster 2, 41 patients (23.6%) as Cluster 3, 39 patients (22.4%) as Cluster 4, 10 patients (5.7%) as Cluster 5, and 21 patients (12.1%) as Cluster 6. The percentage of Elderly and PD patients in the clusters are shown in the right panel of Fig. 2B. Clusters 4, 5, and 6 were considered to be PD-specific clusters due to the small number of Elderly belonging to them. There was no significant difference in the patient's age of each cluster (χ^2^ = 7.988, df = 5, p = 0.16). The duration of disease was significantly longer in Cluster 4 and Cluster 6 than Cluster 1 (χ^2^ = 13.353, df = 5, p = 0.02, Fig. 2B, lower right panel).

The UPDRS results for each cluster are shown in Fig. 2A right panel. The total score was significantly lower in Cluster 6 than Cluster 3 (χ^2^ = 11.37, df = 5, p = 0.04). Regarding the sub-scores, rigidity was significantly higher in Cluster 3 than Clusters 2 and 6, and the rigidity in Cluster 6 was significantly lower than that in Cluster 4 (χ^2^ = 22.384, df = 5, p < 0.01). There was no significant between-cluster difference in bradykinesia (χ^2^ = 8.587, df = 5, p = 0.13), PIGD (χ^2^ = 6.378, df = 5, p = 0.27), or tremor (χ^2^ = 4.249, df = 5, p = 0.51).

**Fig. 3:**
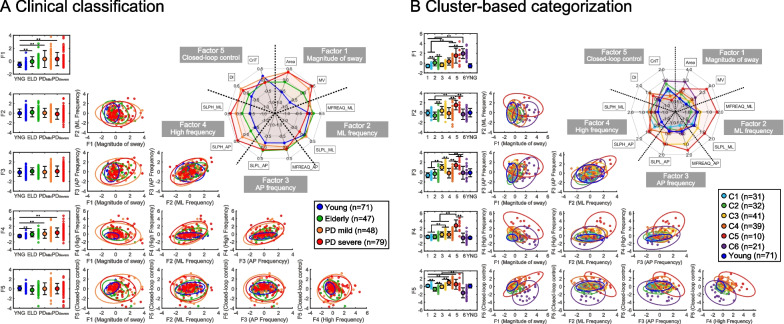
Summary of the results of the exploratory factor analysis. **A** Comparison of factor scores by clinical classification. Data are mean ± SD. *p < 0.05, **p < 0.01 by post-hoc test (Steel–Dwass test) and Kruskal–Wallis test. Scatterplots: the FA in each cluster with 95% confidence ellipsoids and ellipses. Upper right radar chart: the z-scores of the main variables for each factor. The clinical classification shows that each variable has a large overlap in PD severity. **B** Comparison of factor scores by clusters (as in the right part of Fig. 1). Upper right radar chart: the z-scores of the main variables for each factor. For the categories by clusters, the different shapes of the radar chart show the independence and characteristics of each cluster

The results of a detailed comparison of the factor scores and the variables representing factors in each clinical classification (A) and cluster-based categorization (B) are summarized in Fig. 3. The radar chart at the figure's upper right shows the results of the comparison of the variables with the largest contribution of each factor in each group. In the case of clinical classification, the factor scores are largely overlapped among groups. F1 and F4 showed significantly higher values in Elderly, PD_mild_, and PD_severe_ compared to Young (F1: χ^2^ = 45.913, df = 3, p < 0.01, F4: χ^2^ = 43.685, df = 3, p < 0.01). The values were not significantly different in F2 (χ^2^ = 5.224, df = 3, p = 0.16), F3 (χ^2^ = 3.153, df = 3, p = 0.37), and F5 (χ^2^ = 6.908, df = 3, p = 0.07).

In contrast to the clinical classification, the plot of each cluster distributed independently. F1 was significantly lower for Clusters 1 and 3 than for Clusters 4 and 5 (χ^2^ = 85.622, df = 5, p < 0.01). Cluster 1 was also significantly lower than Cluster 2. In contrast, cluster 6 was significantly higher than all clusters excluding cluster 5. In F2, Cluster 5 was significantly higher than the other clusters, and Cluster 3 was significantly higher than Cluster 2 (χ^2^ = 40.304, df = 5, p < 0.01). In F3, Clusters 3 and 5 were significantly higher than Clusters 1, 2, 4, and 6 (χ^2^ = 73.777, df = 5, p < 0.01). In F4, Cluster 5 was significantly higher than the other clusters, and Clusters 3 and 4 were significantly higher than Clusters 1, 2, and 6 (χ^2^ = 47.148, df = 5, p < 0.01). In F5, Clusters 2 and 6 were significantly lower than the other clusters, and Cluster 4 was significantly higher than Clusters 1 and 3 (χ^2^ = 103.9, df = 5, p < 0.01). The detailed statistical results are provided in Table 2.

### Comparison of CoP parameters

**Fig. 4:**
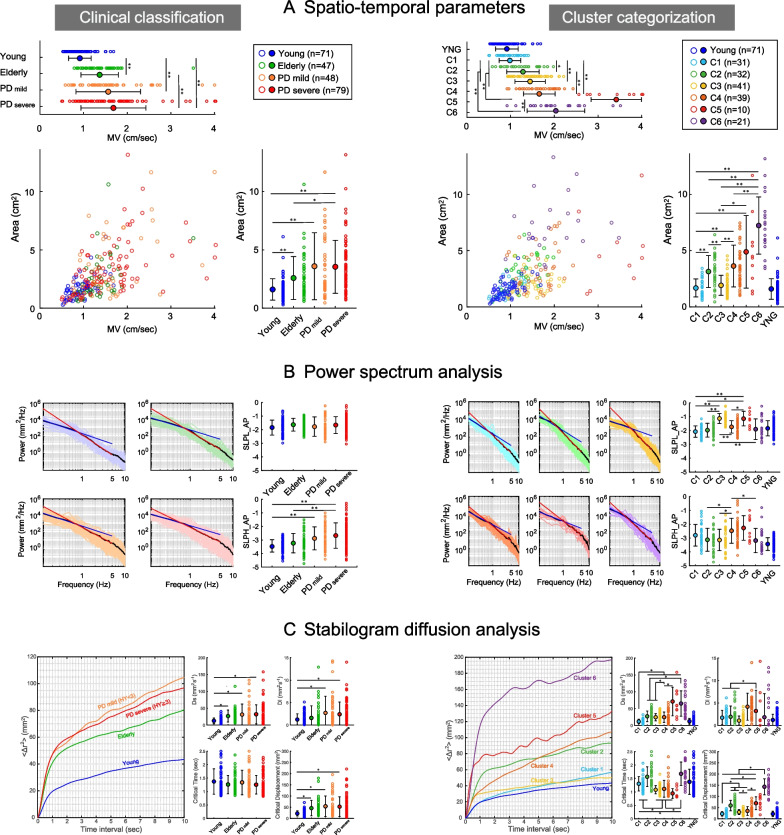
Comparison of CoP parameters by clinical classification and cluster categorization. Left panel: Comparison by clinical classification. Right panel: Comparison of factor scores by clusters. All data are mean ± standard deviation (SD). *p < 0.05, **p < 0.01 by post-hoc test (Steel–Dwass test) in the Kruskal–Wallis test. **A** Comparison of spatiotemporal parameters (Area and MV). **B** Comparison of power-spectrum parameters by anterior–posterior direction (SLPL_AP and SLPH_AP). Each left part: PSD log plot from 0.15 to 10 Hz. The blue line is the regression line for 0.15–1 Hz, and the red line is the regression line for 1–5 Hz. **C** Comparison of results of the stabilogram diffusion analysis (SDA). Each left part: Linear stabilogram diffusion plots in CoP displacements in the planar direction

All evaluated parameters were shown in both the clinical classification (Fig. 4, left panel) and the cluster-based categorization (Fig. 4, right panel). Although the data among the four subject groups (Young, Elderly, PD_mild_, and PD_severe_) widely overlapped, the data in the cluster categorization showed clear separation, which we speculate was due to different contributions of elements comprising the postural instability. Figure 4A shows the data plots of CoP area and mean velocity (MV), which are general spatial and temporal evaluation parameters, respectively. Both the CoP area and MV were significantly higher in the Elderly, PD_mild_, and PD_severe_ groups was compared to the Young group (CoP area: χ^2^ = 46.937, df = 3, p < 0.01, MV: χ^2^ = 77.058, df = 3, p < 0.01). PD_severe_ was also significantly higher than Elderly (p = 0.04). There was no significant difference in either the CoP area or the MV between PD_mild_ and PD_severe_ groups. In the cluster categorization, the CoP area was significantly lower in Clusters 1 and 3 than in other clusters and significantly higher in Clusters 5 and 6 versus the other clusters (χ^2^ = 80.672, df = 5, p < 0.01). The MV was significantly lower in Cluster 1 compared to the other clusters and significantly higher in Cluster 5 than in the other clusters. Cluster 2 was significantly lower than Cluster 4. Cluster 6 was significantly higher than Clusters 1, 2, and 3 (χ^2^ = 85.183, df = 5, p < 0.01).

The logarithmic plots of power spectral density (PSD) by the anterior–posterior direction for each group are demonstrated in Fig. 4B. The clinical classification showed no significant differences in power slopes in the low-frequency band down to 1 Hz (SLPL_AP: χ^2^ = 7.806, df = 3, p = 0.05). In contrast, the slope of power in the high-frequency band above 1 Hz was significantly higher in the PD group (SLPH_AP: χ^2^ = 34.484, df = 3, p < 0.01). In the cluster classification, SLPL_AP was significantly higher in Clusters 3 and 5 than in the other clusters (χ^2^ = 66.017, df = 5, p < 0.01). SLPH_AP was significantly higher in Cluster 4 than in clusters 2, 3, and 6 (χ^2^ = 20.194, df = 5, p < 0.01).

The SDA results for each group and each cluster are summarized in Fig. 4C. As shown by the stabilogram diffusion plot in the figure's left panel, each group and cluster showed clearly different characteristics in both short- and long-term regions. In the clinical classification, Ds, which indicates the expansion of the short-time region, was significantly higher in the Elderly and PD groups than in the Young group (χ^2^ = 46.308, df = 3, p < 0.01). CriD was also significantly higher in the Elderly and PD groups compared to the Young group (χ^2^ = 43.289, df = 3, p < 0.01). In addition, Dl, which indicates the long-time region, was significantly higher in the PD_severe_ group versus the Young group (χ^2^ = 13.41, df = 3, p < 0.01). There was no significant difference in Crit (χ^2^ = 1.852, df = 3, p = 0.60).

In the cluster categorization, Ds was significantly higher in Clusters 5 and 6 than in other clusters, and significantly lower in Cluster 1 than in the other clusters (χ^2^ = 71.736, df = 5, p < 0.01). Dl was significantly higher in Cluster 5 than in Clusters 1, 2, and 3 (χ^2^ = 28.359, df = 5, p < 0.01). CriT was significantly lower in Clusters 3, 4, and 5 versus the other clusters, and significantly higher in Cluster 6 than in Cluster 1 (χ^2^ = 74.211, df = 5, p < 0.01). CriD was significantly higher in Cluster 6 than in the other clusters, and significantly higher in Clusters 2 and 4 than in Clusters 1, 3, and 5 (χ^2^ = 101.03, df = 5, p < 0.01). Cluster 1 was also significantly lower than Cluster 3.

### Comparison of four representative cases

**Fig. 5:**
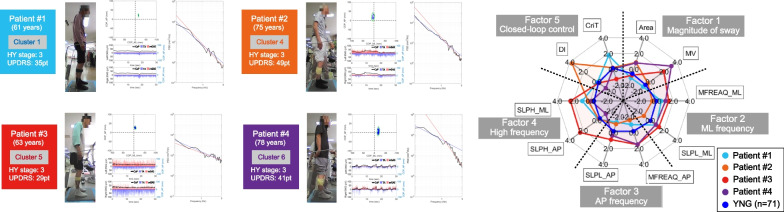
Representative cases. Left panel: The CoP Lissajous figure of CoP, the EMG activity in the gastrocnemius (blue) and tibialis anterior muscles (red), and the power spectral density (PSD) log plot in four representative cases are shown. The blue line in the CoP Lissajous figures shows the trajectory of the CoP, and the green line shows the 95% confidence ellipse. The PSD log plot showed a range from 0.15 to10 Hz. The blue line is the regression line for 0.15–1 Hz, and the red line is the regression line for 1–5 Hz. Right panel: Radar chart of key variables for each factor in four representative cases. The cases are shown as individual Z-scores and Young as mean Z-scores

To validate the interpretation of each cluster, we carried out the characteristics of four representative patients (Fig. 5). Each patient had undergone electromyography (EMG) of the ankle joint (tibialis anterior and gastrocnemius medialis) measured simultaneously with the posturography. Although all four patients were at HY stage 3, they belonged to different clusters and showed different postural control characteristics.

### Patient #1 (Cluster 1)

The patient was a 61-year-old man who had been diagnosed for 3 years. His UPDRS motor score was 35 points, with high scores mainly for tremor, rigidity, and bradykinesia. His postural alignment showed a dropped head, freezing of gait, and easy falling backward in daily life. The postural sway characteristics showed a narrow sway range (F1) and low-frequency characteristics (F2, F3, F4). The tibialis anterior muscle was dominant over the medial gastrocnemius muscle in muscle activity. In this patient's case, the postural sway was smaller than that of healthy young adults, but this may have been due to compensatory postural control for the easy falling backward.

### Patient #2 (Cluster 4)

The patient was a 75-year-old man who had been diagnosed for 3 years. His UPDRS motor score was 49 points, which was high mainly in the scores of rigidity, bradykinesia, and PIGD. He showed decreased flexibility mainly in his trunk. The postural sway characteristics showed a rather wide sway range (F1) but low-frequency characteristics (F2, F3, F4). F5 was high compared to other patients' cases. This patient's muscle activity showed phasic activity of medial gastrocnemius and tibialis anterior muscles with an anterior–posterior CoP shift. Gradual swaying in the low-frequency band was increased due to decreased postural localization by the closed-loop control (F5).

### Patient #3 (Cluster 5)

The patient was a 63-year-old man who had been diagnosed for 9 years. The UPDRS motor score was 29 points, which was high mainly in rigidity, bradykinesia, and PIGD. In daily life, the patient showed easy falling due to freezing of gait. The postural sway characteristics showed a narrow range of sway range (F1) as in Patient #1 but high-frequency characteristics (especially F4). The muscle activity showed co-contraction of the medial gastrocnemius and tibialis anterior muscles. In this patient, the postural sway was narrowed due to the high ankle joint stiffness caused by the co-contraction (shown in F4), suggesting that the postural control was continuous rather than intermittent [40].

### Patient #4 (Cluster 6)

The patient was a 78-year-old man who had been diagnosed for 16 years. His UPDRS motor score was 41 points, and it was high mainly in bradykinesia and PIGD scores. In addition, dyskinesia was observed in the on-medication state. In daily life, the patient was highly active by walking outdoors and engaging in hobby activities, but he experienced frequent falls. The postural sway characteristics showed a wide sway range (F1), high-frequency characteristics (F2, F3, F4), and low F5. The muscle activity showed phasic activity of medial gastrocnemius and tibialis anterior muscles with an anterior–posterior CoP shift. This patient's dyskinesia caused fast and large anterior–posterior swaying, which may have indicated a time delay in the functioning of the closed-loop control.

## Discussion

We attempted to elucidate elements that comprise the postural disturbances in PD patients by conducting an exploratory factor analysis and subsequent cluster analysis based on 23 variables obtained from CoP time-series data during quiet standing. Confirming the clinical significance of the cluster-based categorization, factor loading scores were demonstrated in both the clinical classification (Fig. 3A) and the cluster-based categorization (Fig. 3B). The factor loading scores among the four subject groups (young, elderly, PD_mild_, and PD_severe_) overlapped, whereas those in the cluster categorization showed clear separation which was presumably due to different contributions of the elements of postural instability. Based on the five extracted factors, six distinct subtypes that can be regarded as subtypes of distinct manifestations of postural disorders in patients with PD were identified. In the following section, we first interpret the results of the multivariate analysis and then discuss the clinical importance of the subtype categorization for a better understanding of the postural instability in PD patients.

### Five factors associated with postural control in PD patients

The five factors extracted by the EFA can be reasonably interpreted as the magnitude of sway (F1), ML frequency (F2), AP frequency (F3), high-frequency component (F4), and closed-loop control (F5). The ordinary clinical assessment of postural instability using CoP data (so-called posturography) is evaluated using mainly spatio-temporal variables and frequency characteristics [33, 41]. The magnitude of the sway, frequency components, and the direction of the sway have also been detected in a principal component analysis (PCA) using the CoP data of PD patients [42]. Our present findings also demonstrated that the factor loading score of the high-frequency component (F4) in the elderly and PD patients was significantly higher than that in the healthy young subjects. This is in agreement with the previous finding that the high-frequency component is one of the clinical manifestations in elderly and PD patients [12, 43].

The high-frequency component may directly reflect the problem of postural instability [44] and reflects elements of postural tremor in PD, especially in the off-medication state [12]. In the present study, the frequency response below 1 Hz, explained by the integration of F2 and F3, was not affected by the severity of PD. The magnitude of the standard deviation in each factor score of the PD patients further clarified the presence of subtypes in postural instability.

F5 could be interpreted as relevant to closed-loop control because this factor had a higher loading score with the SDA-related variables. Dl is related to diffusion in closed-loop functions and had a strong negative correlation with joint stiffness in model simulation studies [45], and CriT indicates the time interval for stability maintenance as determined by the closed-loop system. Taken together with these facts, we speculate that F5 might reflect the extent of time delay in the closed-loop system and/or reduced stiffness.

As shown in Fig. 3, the clear separation of the distribution of each factor loading score suggests that a factor analysis can reliably extract elements comprising postural control in PD patients. The factor analysis process was designed to break down postural instability into coherent profiles among 23 parameters, and we were then able to successfully extract distinct five components of postural control strategy. Considering the complex interaction of elements of postural instability in PD, we speculate that certain types of postural control strategy consist of different contributions of each of the five components.

### Subtypes of postural disturbance revealed by cluster analysis

The GMM-based cluster analysis based on the obtained factor loading scores was able to identify subtypes of postural disturbance/instability in PD patients. In contrast to the clinical classification, the cluster-based categorization enabled us to re-group the distinct types of postural instability. More importantly, it is meaningful to discuss the contribution of each of the five factors to each cluster for a better understanding of PD-related postural disturbance.

Cluster 1 had the shortest post-onset period and was similar to the young healthy group in each factor score except for the frequency component (F3, F4). Therefore, Cluster 1 can be interpreted as mild postural instability. However, almost half of the subjects in Cluster 1 belonged to the PD_severe_ group. This may have included patients with excessive narrowing of postural sway, such as Patient #1 presented as a representative case. Cluster 2 was characterized by higher loading of F1 and lower loading of F5 compared to Cluster 1. This also reflects the profile of SDA, which showed larger Ds and higher Crit values, suggesting a delay in the time interval of the closed-loop system. Cluster 3 was characterized by lower loading of F1, but higher loading of F2 and F3, suggesting that the range of sway is narrow but the frequency component is different from Cluster 1 (larger SLPL_AP and higher Ds in Cluster 3 than Cluster 1). Such characteristics might have relevance to the highest rigidity score in UPDRS. Concerning this point, another study suggested that a narrow range of postural sway can be attributed to rigidity due to malfunction of the control of muscle stiffness [46].

Cluster 4 showed similar scores to Cluster 1 in many factor scores, but F5 showed the highest scores. As clearly indicated by the higher Dl, the patients categorized in Cluster 4 had attenuated closed-loop control with a large range of postural sway during upright standing. Cluster 5 was characterized by higher loading of F2 and F3, which is similar to Cluster 3, but it had significantly higher loading of F4 than the other clusters. High F4 was suggested to be controlled by co-contraction of overactivity of the ankle muscles, as shown in the typical data of Patient #2. Such cases may represent a continuous control model deviating from intermittent control [40, 47]. Regarding the high F1, it may reflect the influence of MV, which is strongly associated with high-frequency components (see the radar chart in Fig. 4C).

Cluster 6 showed higher F1 and lower F5, suggesting greater sway and a delay in the time interval to the closed-loop system function. The large Ds and Crit values in SDA may reflect lower loop gains in the postural control model [48]. Interestingly, Cluster 6 showed the lowest UPDRS total and rigidity scores compared to the other clusters. This result is consistent with studies that found no relationship between the UPDRS and the magnitude of sway during quiet standing [12, 49]. Cluster 6 also had a longer time since onset and a younger age of onset. This may indicate a more slowly progressing disease type of PD with milder motor symptoms [50]. PD patients who have been on-medication for a long time also often have dyskinesia problems [51]. PD patients with dyskinesia have been reported to have increased sway velocity and an increased total path length [52, 53]. In fact, Patient #4 in Cluster 6 showed dyskinesia in the on-medication state. The large F1 shown in Cluster 6 may reflect elements of dyskinesia.

### The clinical importance of the cluster-based subtype categorization

It is clear that the commonly used parameters of CoP alone, i.e., sway area and velocity, are insufficient to characterize postural instability. It is important to focus on the selection of the optimal evaluation parameters and the most suitable analysis process. We were motivated to characterize postural instability in PD patients based on a multivariate analysis using CoP data recording during quiet standing in healthy young, elderly, and PD patients. We performed a factor analysis of 23 variables calculated using the subjects' CoP in order to extract distinct components underlying postural instabilities in PD patients. This analysis process enabled us to understand the characteristics and elements that comprise postural instability in PD patients. As shown in Fig. 1, the results of the factor analysis revealed distinct five elements of postural disturbance in PD patients; a similar analysis process was used to characterize gait behavior [54] and visuospatial cognition [55] in studies that effectively compressed the data and then identified distinct subtypes.

Another important task is to elucidate the complex elements of postural disturbance. The clear separation observed in this study was largely affected by the methodological constraints of the multivariate analysis which aimed to reveal distinct separation of the target behavior, and further comparisons of the clinical classification and cluster-based categorization would give us more important information for clinical evaluations. That is, the problem of insufficiency in the detection of differences between patient populations is not simply due to the evaluation method and parameters; rather, the complexity of the elements comprising neurological behavior is also involved.

It can be emphasized that the process of cluster-based categorization is a type of causal inference of postural instability. As illustrated in Fig. 4, almost all of the CoP-related evaluated parameters and the SDA profile did not show clear differences among the elderly, PD_mild_, and PD_severe_ groups. On the other hand, each cluster showed clear separation in most of the parameters, which is presumably due to different contributions of the elements underlying postural instability.

### Implications, limitations, and future direction

With regard to the evaluated parameters, the commonly used spatio-temporal parameters can be intuitive for evaluating the magnitude of the postural sway/disturbance of patients. Nevertheless, it is important to pay attention to the manner of control behind the postural behavior. A SDA is one of the established fractal time-series analysis of the variability of the sum of the squares of the distances between the start and end points of a motion over different time ranges. Collins and De Luca reported that the behavior of the CoP during quiet standing is characterized by distinct two components revealed by an SDA analysis, namely, the profile of short- and long-time intervals [34]. Those authors speculated that the CoP shows a spontaneous drift away from the relative equilibrium point and persists (i.e., control in an open-loop system) during short time intervals, whereas during long time intervals the CoP behavior is anti-persistent and returns to the relative equilibrium point (i.e., control in a closed-loop system). The transition between the short- and long-time regions is interpreted as the critical point in the average time interval and its displacement where the attitude control switches from an open-loop system to a closed-loop system. Analyses of postural control characteristics using an SDA can provide insight into the different control mechanisms of the diverse postural instabilities presented by PD patients. While a SDA is not commonly used in clinical evaluations, the SDA profile in each cluster in the present study clearly demonstrated different contributions of short- and long-time interval regions and its contribution.

Since the present study focused on the mechanisms underlying postural control we could not directly discuss about relevance between postural instability and risk of fall in PD patients. In the future study, we should include whole-body kinematics measurements to achieve a more robust understanding of postural instability in PD patients and then should discuss the above-mentioned point. Differences in postural alignment are expected to have a significant impact on postural control [56], such as Camptocormia and Pisa syndrome [57]. The effect of medication (on/off status) on postural instability should also be clarified with the use of the clusters detected in this study.

## Conclusions

The present study was conducted to identify the components of postural control in PD patients by EFA using various evaluation parameters obtained from CoP time series data during quiet standing. Since the extracted factors obtained from EFA were the result of data compression and multivariate analysis, those can be regarded as consisting elements of postural control in PD patients. Cluster classification based on factor scores clearly identified symptom-dependent differences among PD patients. Such subtype classification provide useful information for the better understanding of postural disorders in PD patients and give clinicians and therapist to prescribe appropriate rehabilitation intervention based on the clinical manifestation and the type of postural disorders.

## Data Availability

The data and codes used in this study are available on request, in anonymized format, from the corresponding author. Information and requests for resources should be directed to and will be fulfilled by the corresponding author, Noritaka Kawashima (kws456123@gmail.com).
